# Increasing n-butanol production with *Saccharomyces cerevisiae* by optimizing acetyl-CoA synthesis, NADH levels and trans-2-enoyl-CoA reductase expression

**DOI:** 10.1186/s13068-016-0673-0

**Published:** 2016-11-25

**Authors:** Virginia Schadeweg, Eckhard Boles

**Affiliations:** Institute of Molecular Biosciences, Goethe-University Frankfurt, Max-von-Laue Str.9, 60438 Frankfurt am Main, Germany

**Keywords:** n-Butanol, *Saccharomyces*, Coenzyme A, Acetyl-CoA, Pantothenate, Acetylating acetaldehyde dehydrogenase, Trans-2-enoyl-CoA reductase

## Abstract

**Background:**

n-Butanol can serve as an excellent gasoline substitute. Naturally, it is produced by some *Clostridia* species which, however, exhibit only limited suitability for industrial n-butanol production. The yeast *Saccharomyces cerevisiae* would be an ideal host due to its high robustness in fermentation processes. Nevertheless, n-butanol yields and titers obtained so far with genetically engineered yeast strains are only low.

**Results:**

In our recent work, we showed that n-butanol production via a clostridial acetoacetyl-CoA-derived pathway in engineered yeast was limited by the availability of coenzyme A (CoA) and cytosolic acetyl-CoA. Increasing their levels resulted in a strain producing up to 130 mg/L n-butanol under anaerobic conditions. Here, we show that under aerobic conditions. this strain can even produce up to 235 mg/L n-butanol probably due to a more efficient NADH re-oxidation. Nevertheless, expression of a bacterial water-forming NADH oxidase (nox) significantly reduced n-butanol production although it showed a positive effect on growth and glucose consumption. Screening for an improved version of an acetyl-CoA forming NAD^+^-dependent acetylating acetaldehyde dehydrogenase, adhE^A267T/E568K/R577S^, and its integration into n-butanol-producing strain further improved n-butanol production. Moreover, deletion of the competing NADP^+^-dependent acetaldehyde dehydrogenase Ald6 had a superior effect on n-butanol formation. To increase the endogenous supply of CoA, amine oxidase Fms1 was overexpressed together with pantothenate kinase coaA from *Escherichia coli*, and could completely compensate the beneficial effect on n-butanol synthesis of addition of pantothenate to the medium. By overexpression of each of the enzymes of n-butanol pathway in the n-butanol-producing yeast strain, it turned out that trans-2-enoyl-CoA reductase (ter) was limiting n-butanol production. Additional overexpression of ter finally resulted in a yeast strain producing n-butanol up to a titer of 0.86 g/L and a yield of 0.071 g/g glucose.

**Conclusions:**

By further optimizing substrate supply and redox power in the form of coenzyme A, acetyl-CoA and NADH, n-butanol production with engineered yeast cells could be improved to levels never reached before with *S. cerevisiae* via an acetoacetyl-CoA-derived pathway in synthetic medium. Moreover, our results indicate that the NAD^+^/NADH redox balance and the trans-2-enoyl-CoA reductase reaction seem to be bottlenecks for n-butanol production with yeast.

**Electronic supplementary material:**

The online version of this article (doi:10.1186/s13068-016-0673-0) contains supplementary material, which is available to authorized users.

## Background

Butanol isomers like n-butanol or isobutanol are regarded as more suitable fuel substitutes than bioethanol. Butanol production via genetically engineered yeast cells has recently been reviewed in [1]. In our previous work, we have engineered the yeast *Saccharomyces cerevisiae* for n-butanol production via a *Clostridia*-derived acetoacetyl-CoA-dependent pathway [2] (Additional file 1: Figure S1). It turned out that the availability of coenzyme A (CoA) and cytosolic acetyl-CoA are limiting heterologous n-butanol production with yeast. For industrial processes, the yeast *S. cerevisiae* is established as a highly robust, easily genetically modifiable, well-characterized and phage infections-resistant organism for fermentation [3]. However, in *S. cerevisiae,* the acetyl-CoA metabolism takes place in four different compartments, which hampers metabolic engineering for certain products. Acetyl-CoA functions as a precursor for several metabolic pathways in yeast. It is the end product of fatty acid β-oxidation, a precursor for energy generation, but at the same time, a substrate for fatty acid, isoprenoid, and amino acid biosynthesis. Therefore, acetyl-CoA is a favorable starting point for biotechnological applications which focus on production of lipids, polyketides, isoprenoids, and alcohols [4].

Acetyl-CoA is present in the cytosol, mitochondria, nucleus, and peroxisomes in baker’s yeast. Cytosolic acetyl-CoA is produced via the pyruvate dehydrogenase (PDH)-bypass from pyruvate which first is converted into acetaldehyde via pyruvate decarboxylases. Acetaldehyde is then transformed into acetate mainly by acetaldehyde dehydrogenase Ald6, which uses NADPH as cofactor. Finally, acetate is converted into acetyl-CoA via ATP-consuming acetyl-CoA synthetases (ACSs). However, acetaldehyde is mainly diverted to ethanol, which is the main reduction product in yeast. Also, the other precursors of acetyl-CoA, pyruvate or acetate, can enter into one of the other mentioned compartments, and therefore less acetyl-CoA is available in cytosol [4]. Besides, there do not exist direct transport systems for acetyl-CoA between the compartments but only the glyoxylate cycle or carnitine/acetyl-carnitine shuttle [5].

Also, the Crabtree effect limits the availability of cytosolic acetyl-CoA. Most of glucose is converted into ethanol, even under aerobic conditions [6]. Another byproduct is glycerol, whose reduction pathway is also used for NAD^+^ regeneration to enable further glucose oxidation in glycolysis. To increase cytosolic acetyl-CoA levels for biotechnological purposes, several approaches have been undertaken. Alcohol dehydrogenase genes (ADH) were deleted to limit ethanol production; enzymes of the PDH-bypass were overexpressed [4]; ATP-independent heterologous PDH complexes were expressed in the yeast cytosol [7]; or likewise, ATP-independent, heterologous pyruvate formate lyase was expressed [7, 8]. Moreover, acetylating acetaldehyde dehydrogenases were tested, which convert acetaldehyde directly into acetyl-CoA without ATP consumption. The functionality of these enzymes in yeast was proven by overexpression in *acs2* deletion mutants, as *acs2* mutants are not able to grow on glucose without expressing another enzyme producing acetyl-CoA in the cytosol [7].

A further promising enzyme is *adhE* from *E.coli*, which is a multifunctional oxidoreductase. This enzyme consists of two domains, an acetaldehyde oxidoreductase and an ethanol oxidoreductase, and is probably an evolutionary product of a gene fusion. A reason for this might be that catalytic sites are now closer together and one NADH binding site is shared [9]. In *E.coli,* adhE catalyzes the reduction from acetyl-CoA into acetaldehyde and then into ethanol. However, Membrillo-Hernandez and coworkers were able to engineer the enzyme to prefer the conversion of acetaldehyde into acetyl-CoA. For that, two point mutations were introduced: A267T and E568K. Mutation A267T was mainly responsible for the improved catalytic activity, and E568K stabilized the architectural integrity [9]. Thus, adhE^A267T/E568K^ can function as an acetylating acetaldehyde dehydrogenase, as well.

**Fig. 1:**
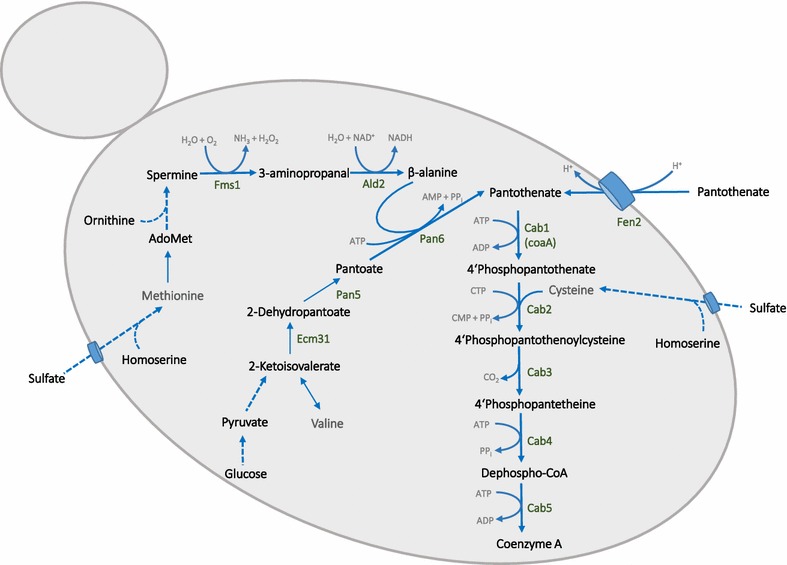
Simplified metabolic pathway for CoA biosynthesis in *Saccharomyces cerevisiae.* Shown are the relevant steps in yeast’s metabolism for endogenous synthesis of free CoA. The precursor pantothenate can be taken from medium via transporter Fen2 or can be synthesized endogenously from amino acids

Independent of the use of the endogenous PDH-bypass or heterologous enzyme for cytosolic acetyl-CoA production, free CoA is also needed. Biosynthesis of CoA starts from pantothenate in yeast (Fig. 1). First, pantothenate is converted into 4′-phosphopantothenate by pantothenate kinase Cab1. Together with cysteine, 4′-phosphopantothenate is then transformed into 4′-phosphopantothenoylcysteine, which is further converted into 4′-phosphopantetheine, dephospho-CoA, and finally CoA [10]. The pantothenate kinase reaction is limiting CoA synthesis in yeast and is inhibited by acetyl-CoA [10, 11]. Therefore, we used the overexpression of pantothenate kinase coaA from *E.coli* to increase CoA and n-butanol synthesis in yeast [2].

Additionally, in order to enhance CoA and finally acetyl-CoA production in the cytosol, increased supply of pantothenate in the growth medium is necessary [2]. Yeast cells can either take up pantothenate from the medium via the Fen2 transporter [12] or can synthesize it via an endogenous pathway starting from amino acids methionine and valine (Fig. 1). Valine is transaminated into 2-ketoisovalerate, which serves as a precursor for pantoate production. Pantoate in turn is transformed with β-alanine into pantothenate in an ATP-dependent reaction. The precursor of β-alanine is methionine, which is first converted to spermine [13]. The next step, the conversion of spermine into 3-aminopropanal via amine oxidase Fms1, was found to be rate limiting for pantothenate synthesis [14]. Indeed, overexpression of *FMS1* even led to pantothenate excretion into the media. Further on, 3-aminopropanal is transformed into β-alanine by aldehyde dehydrogenases Ald2/3, whereby Ald2 is mainly responsible for this reaction [13].

Heterologous n-butanol production via the acetoacetyl-CoA-derived pathway in engineered yeasts needs high levels of acetyl-CoA [2, 6]. In this pathway, two molecules of acetyl-CoA are condensed to acetoacetyl-CoA. This reaction is catalyzed by endogenous yeast thiolase Erg10 [15]. Then, acetoacetyl-CoA can be reduced into 3-hydroxybutyryl-CoA and further converted into crotonyl-CoA via heterologous dehydrogenases and crotonases like hbd and crt from *C. acetobutylicum* [16]. For the irreversible conversion of crotonyl-CoA into butyryl-CoA, trans-2-enoyl-CoA reductase ter of *Treponema denticola* was shown to be useful [6]. The last steps from butyryl-CoA into butyraldehyde and n-butanol can be catalyzed by aldehyde dehydrogenase eutE from *E. coli* [17] and/or aldehyde/alcohol dehydrogenase adhE2 from *C. acetobutylicum* [18]. Furthermore, on rich medium or via engineering of amino acid synthesis and degradation, *S. cerevisiae* is able to produce n-butanol also by endogenous amino acid-derived pathways (reviewed in [1]). In a recent study, the endogenous pathway of threonine was optimized and combined with an introduced citramalate synthase-mediated pathway, which enabled n-butanol production of 835 mg/L in complex medium under anaerobic conditions [19].

So far, with the contribution of only the acetoacetyl-CoA-derived pathway in synthetic medium without addition of amino acids, metabolic engineering of *S. cerevisiae* resulted in n-butanol titers from 2.5 mg/L [20] up to 130 mg/L under anaerobic conditions [2]. Here, we report further improvements for n-butanol production with *S. cerevisiae* via an acetoacetyl-CoA-derived pathway under aerobic conditions.

## Methods

### Strains and media

Yeast strains used in this study are listed in Table 1. Media composition was as described in [2]. Ethanol concentration in liquid and solid media was 2% (v/v). For cross-feeding assays on solid synthetic complete medium (SCD) lacking pantothenate, 1.7 g/L yeast nitrogen base without pantothenate (YNB-Calcium pantothenate) was used from the company Sunrise Science Products, San Diego, CA 92131. Moreover, SCD medium contained 5 g/L ammonium sulfate, amino acid supplements, as well as 7.5 g/L agar for soft agar (standard agar with 20 g/L) and was adjusted to pH 6.3 with potassium hydroxide.

**Table 1:** Yeast strains and plasmids used in this study

Strain or plasmid	Characteristics	Reference
Strains		
VSY0	*MATa; ura3*-*52; trp1*-*289; leu2*-*3_112; his3*Δ*1; MAL2*-*8C; SUC2* *adh1::loxP adh3::loxP adh5::loxP adh4*Δ*::loxP adh2*Δ*::LEU2*	[2]
Y00000	BY4741 *MATa; his3*Δ*1; leu2*Δ*0; met15*Δ*0; ura3*Δ*0*	Euroscarf, Frankfurt
Y00595	BY4741 *Mata; his3*Δ*1; leu2*Δ*0; met15*Δ*0; ura3*Δ*0; fms1::kanMX4*	Euroscarf, Frankfurt
Y02304	BY4741 *MATa; his3*Δ*1; leu2*Δ*0; met15*Δ*0; ura3*Δ*0;* *pan6::kanMX4*	Euroscarf, Frankfurt
Y06868	BY4741 *MATa; his3*Δ*1; leu2*Δ*0; met15*Δ*0; ura3*Δ*0;* *acs1::kanMX4*	Euroscarf, Frankfurt
JDY2	*MATα; his3*Δ*1; leu2*Δ*0; lys2*Δ*0; MET15; ura3*Δ*0; acs2::kanMX4*	Lab stocks, Boles group
VSY4	*MATa; his3*Δ*1; leu2*Δ*0; met15*Δ*0; ura3*Δ*0; acs1::kanMX4 sfa1*Δ: ^Ec^ *adhE* ^A267T/E568K^/*hphNT1*	This work
VSY5	*MATα; his3*Δ*1; leu2*Δ*0; lys2*Δ*0; MET15; ura3*Δ*0; acs2::kanMX4;* *sfa1*Δ: ^Ec^ *adhE* ^A267T/E568K^, *hphNT1*	This work
VSY7	*MATα; his3*Δ*1; leu2*Δ*0; lys2*Δ*0; MET15; ura3*Δ*0; acs2*Δ:^Ec^ *adhE* ^A267T/E568K^, *hphNT1; acs1::kanMX4*	This work
VSY7_evolved	*MATα; his3*Δ*1; leu2*Δ*0; lys2*Δ*0; MET15; ura3*Δ*0; acs2*Δ:^Ec^ *adhE* ^A267T/E568K/R577S^, *hphNT1; acs1::kanMX4* with unknown mutations for growth on glucose	This work
VSY7_R577S	*MATα; his3*Δ*1; leu2*Δ*0; lys2*Δ*0; MET15; ura3*Δ*0; acs2*Δ:^Ec^ *adhE* ^A267T/E568K/R577S^, *hphNT1; acs1::kanMX4*	This work
VSY10	*MATa; ura3*-*52; trp1*-*289; leu2*-*3_112; his3*Δ*1; MAL2*-*8C; SUC2* *adh1::loxP; adh3::loxP: adh5::loxP; adh4*Δ*::loxP; adh2*Δ*::LEU2; sfa1*Δ: ^Ec^ *adhE* ^A267T/E568K^ *, hphNT1; adh6*Δ: ^Ec^ *coaA, natNT2; gpd2*Δ: ^Sc^ *ERG10,* ^Ca^ *hbd,* ^Ca^ *crt,* ^Td^ *ter,* ^Ca^ *adhE2,* ^Ec^ *eutE, kanMX*	[2]
VSY13	*MATa; ura3*-*52; trp1*-*289; leu2*-*3_112; his3* Δ*1; MAL2*-*8C; SUC2* *adh1::loxP; adh3::loxP; adh5::loxP; adh4*Δ*::loxP; adh2*Δ*::LEU2; adh6*Δ: ^Ec^ *coaA, loxP; sfa1*Δ: ^Ec^ *adhE* ^A267T/E568K/R577S^ *, loxP; gpd2*Δ: ^Sc^ *ERG10,* ^Ca^ *hbd,* ^Ca^ *crt,* ^Td^ *ter,* ^Ca^ *adhE2,* ^Ec^ *eutE, kanMX*	This work
VSY15	*MATa; ura3*-*52; trp1*-*289; leu2*-*3_112; his3* Δ*1; MAL2*-*8C; SUC2* *adh1::loxP; adh3::loxP; adh5::loxP; adh4*Δ*::loxP; adh2*Δ*::LEU2; adh6*Δ: ^Ec^ *coaA, loxP; sfa1*Δ: ^Ec^ *adhE* ^A267T/E568K/R577S^ *, loxP;* pFMS1Δ*::HIS3,* pADH1; *gpd2*Δ: ^Sc^ *ERG10,* ^Ca^ *hbd,* ^Ca^ *crt,* ^Td^ *ter,* ^Ca^ *adhE2,* ^Ec^ *eutE, kanMX*	This work
VSY19	*MATa; ura3*-*52; trp1*-*289; leu2*-*3_112; his3* Δ*1; MAL2*-*8C; SUC2* *adh1::loxP; adh3::loxP; adh5::loxP; adh4*Δ*::loxP; adh2*Δ*::LEU2; adh6*Δ*:: coaA, loxP; sfa1*Δ*:: adhE* ^A267T/E568K/R577S^ *, loxP;* pFMS1Δ*::HIS3,* pADH1; *ald6*Δ; *gpd2*Δ: ^Sc^ *ERG10,* ^Ca^ *hbd,* ^Ca^ *crt,* ^Td^ *ter,* ^Ca^ *adhE2,* ^Ec^ *eutE, kanMX*	This work
Plasmids		
pRS41H	CEN6ARS4*, hphNT1, Ampr*	[29]
pRS62H	2µ*, hphNT1, Ampr,* shortened *HXT7* promoter and *CYC1* terminator	[30]
pVS4	CEN6ARS4, *hphNT1*, Ampr, ^Ec^ *adhE* ^A267T/E568K^	[2]
pVS4ev	CEN6ARS4, *hphNT1*, Ampr, ^Ec^ *adhE* ^A267T/E568K/R577S^	This work
pRCC-K_ALD6	2µ, *kanMX, Ampr,* pROX3_*Cas9_*tCYC1, gRNA for *ALD6*	This work
pRS62H_ERG10	2µ*, hphNT1, Ampr,* ^Sc^ *ERG10*	This work
pRS62H_hbd	2µ*, hphNT1, Ampr,* ^Ca^ *hbd*	This work
pRS62H_crt	2µ*, hphNT1, Ampr,* ^Ca^ *crt*	This work
pRS62H_ter	2µ*, hphNT1, Ampr,* ^Td^ *ter*	This work
pRS62H_adhE2	2µ*, hphNT1, Ampr,* ^Ca^ *adhE2*	This work
pRS62H_eutE	2µ*, hphNT1, Ampr,* ^Ec^ *eutE*	This work
pRS41H_pTPI1_NOX	CEN6ARS4*, hphNT1, Ampr,* NADHoxidase ^Sp^ *NOX*	Lab stocks, Boles group

Genes from Sc, *Saccharomyces cerevisiae*; Ca, *Clostridium acetobutylicum*; Ec, *E.coli*; Td, *Treponema denticola*; Sp, *Streptococcus pneumoniae* are indicated by prefixes in superscript. Promoters are indicated in Additional file 1: Table S2. *kanMX* G418 resistance, *hphNT1* hygromycin resistance, *Ampr* ampicillin resistance

### Plasmid and strain construction

Assembly of vectors and constructions of VSY0 (Δ*adh1*–*5*) and VSY10 were described in [2], and important metabolic routes are shown in Additional file 1: Figure S1. All genes were codon-optimized according to the yeast glycolytic codon usage [21], except for ^Sc^ *ALD2* and ^Sc^ *PAN6*, which were amplified from chromosomal DNA. Strains VSY4, 5, and 7 were established in order to evolve and compare strains with Δ*acs1* or/and Δ*acs2* deletion carrying ^Ec^ *adhE* ^A267T/E568K^ (Table 1). Therefore, ^Ec^ *adhE* ^A267T/E568K^ with promoter *pPFK1* and terminator *tDIT1* was integrated via homologous recombination with 400 bp overlaps into the respective gene locus on YEPD or YEPE in corresponding BY strains (Y06868 and JDY2). Acetylating acetaldehyde dehydrogenase was introduced in the already deleted *ACS2* locus of JDY2, and then *ACS1* was deleted with *kanMX* deletion cassette. Resulting VSY7 was grown aerobically in 30 mL YEPD with a starting OD_600_ of 0.3 for 8 days until an OD_600_ of 2 was reached. In a next round under the same conditions, an OD_600_ of 2 was reached already after 1 day (VSY7_evolved). Sequencing of ^Ec^ *adhE* ^A267T/E568K^ locus of one single clone revealed a new mutation, and ^Ec^ *adhE* ^A267T/E568K/R577S^ was amplified from chromosomal DNA in order to introduce it in VSY7. The resulting strain was named VSY7_R577S.

In VSY10 ^Ec^ *adhE* ^A267T/E568K^ was exchanged against ^Ec^ *adhE* ^A267T/E568K/R577S^, which resulted in VSY13. Furthermore, the native promoter of *FMS1* (300 bp upstream) was exchanged against the strong glycolytic promoter *pADH1* with the aid of *HIS3* marker. Fragments containing 400 bp homologous region, *HIS3* sequence, *pADH1,* and 400 bp homologous region of *FMS1* were assembled via fusion PCR [22] in order to integrate the cassette into VSY13, resulting in VSY15. For further deletion of *ALD6* in VSY15, CrisprCas method was used [23] (crRNA was generated by www.dna20.com), resulting in VSY19. Genome integrations and deletions were confirmed by PCR analysis. All primers and used donors for CrisprCas system are shown in Additional file 1: Table S1 and promoters and terminators of cloned genes in Additional file 1: Table S2.

### Fermentations for n-butanol production

All fermentation conditions, as well as HPLC measurements, were described in [2]. One difference was that fermentations were carried out aerobically in 30 mL SMD media and not semi-anaerobically and that additionally, potassium acetate was used as a standard for HPLC with concentrations of 0.5–20 g/L.

### Growth assays and cross-feeding tests

For growth assays on solid media, cultures were grown to exponential phase, washed, and adjusted to an OD_600_ of 1. Three tenfold serial dilutions were prepared, and 5 µl of each solution was spotted on YEPD, YEPE, or SCD agar plates and incubated at 30 °C under aerobic conditions. For cross-feeding tests on solid media [14], 10 µl of overnight cultures of Δ*fms1* or Δ*pan6* were added to SCD agar without pantothenate.

### Enzyme assay

For enzyme assays, Δ*adh1*–*5* (VSY0) was transformed with respective vector (pRS41H, pVS4, pVS4ev) and incubated overnight in 50 mL YEPD to an OD of OD_600_ 0.8–1. Further preparation and implementation of enzyme assays were conducted as described in [2].

For dehydrogenase activity of the acetylating acetaldehyde dehydrogenase (*adhE* ^A267T/E568K^), the reaction buffer contained 50 mM CHES and 0.2 mM DTT with pH 9.5. The increase of NADH concentrations was monitored at 340 nm. The acetaldehyde dehydrogenase activity was measured with 20 mM acetaldehyde as substrate and 0.8 mM NAD^+^ and 0.1 mM CoA as cofactors. The reaction was started by the addition of cooled acetaldehyde (based on [7]). Stability assay was performed by preparing aliquots of cell extract, that were kept on ice until incubation at 30° in a thermo block. Dehydrogenase activity assay was directly performed after incubation for 0–40 min at 30° (based on [9]).

## Results and discussion

### Aerobic conditions improve n-butanol production in engineered n-butanol-producing *S. cerevisiae*

In our previous work [2] we had constructed a yeast strain engineered with an acetoacetyl-CoA-derived n-butanol production pathway and optimized for enhanced CoA and acetyl-CoA synthesis, strain VSY10. Fermentations with this strain were performed under semi-anaerobic oxygen-limited conditions and resulted in n-butanol titers up to 130 mg/L and yields up to 0.012 g/g glucose. Nevertheless, VSY10 was not able to consume more than about half of the glucose. This might be due to inefficient NADH re-oxidation under the anaerobic conditions as ethanol and glycerol production were nearly eliminated in the strain and the introduced n-butanol pathway was obviously not yet strong enough to compensate these deficiencies.

Under aerobic conditions, cytosolic NADH can be re-oxidized also by external mitochondrial NADH dehydrogenases or by mitochondrial redox shuttle mechanisms [24]. Therefore, we repeated the fermentations with VSY10 now under more aerobic conditions with shake flask cultures. The fermentations were performed in SMD media supplemented with additional 25 µM pantothenate to enhance CoA synthesis [2].

Indeed, glucose consumption of VSY10 was improved under aerobic conditions (Additional file 1: Figure S2a), and n-butanol titers increased up to 235 mg/L (Table 2). Moreover, growth was improved to final OD_600_ values of more than 2 (Additional file 1: Figure S2b), whereas under anaerobic conditions, OD_600_ values did not exceed 1.8. Ethanol production did not change substantially (Additional file 1: Figure S3). Nevertheless, due to the higher glucose consumption, n-butanol yields were only slightly increased under aerobic conditions (Table 2) and reached 0.013 g/g glucose.

**Table 2:** n-Butanol titers and yields of n-butanol producing strains

Strain	n-Butanol	
	Titer [mg/L]	Yield [g/g glucose]
Anaerobic		
VSY10	108.08 ± 10.8	0.010 ± 0.0003
VSY10 + 25 µM pantothenate	130.46 ± 19.7	0.012 ± 0.0008
Aerobic		
VSY10	148.21 ± 14.08	0.012 ± 0.0005
VSY10 + 25 µM pantothenate	235.02 ± 7.74	0.013 ± 0.0005
VSY13	165.00 ± 15.42	0.010 ± 0.0017
VSY13 + 25 µM pantothenate	247.34 ± 8.23	0.015 ± 0.0006
VSY15	243.20 ± 14.13	0.016 ± 0.0017
VSY15 + pVS4ev	254.49 ± 5.89	0.017 ± 0.0001
VSY15 + pRS62H_tdTer	448.72 ± 3.16	0.028 ± 0.0010
VSY19	633.92 ± 19.33	0.057 ± 0.002
VSY19 + pVS4ev	643.31 ± 17.42	0.047 ± 0.0007
VSY19 + pRS62H_tdTer	859.05 ± 120.32	0.071 ± 0.006

Titers and yields of anaerobic [1] and aerobic fermentations in SMD with or without addition of 25 µM pantothenate and with or without additional pVS4ev or pRS62H_tdTer vector are shown for strains VSY10 (Δ*adh1*–*6 sfa1 gpd2*, with n-butanol pathway genes, *coaA* and *adhE* ^A267T/E568K^), VSY13 (like VSY10, but *adhE* ^A267T/E568K/R577S^ instead of *adhE* ^A267T/E568K^), VSY15 (VSY13 with pADH1_*FMS1*) and VSY19 (VSY15 with Δ*ald6*). The maximal titers in fermentations are shown, which is for VSY10 and VSY15/VSY19 + pRS62H_tdTer after 74 h and VSY19 strains after 50 h and for all other strains after 100 h. Yields are always calculated with values referring to samples taken after 74 or 50 h. The mean values of three independent replicates are shown with standard deviations

### A R577S mutation in adhE increases acetyl-CoA supply and further improves n-butanol production

In VSY10, ATP-independent acetyl-CoA synthesis had been increased via introduction of an acetylating acetaldehyde dehydrogenase mutant enzyme from *E. coli*, adhE^A267T/E568K^ [2]. This mutant adhE form favors the conversion of acetaldehyde to acetyl-CoA instead of converting acetyl-CoA to ethanol (Additional file 1: Figure S4) [9] but only if enough free CoA is available [2]. Nevertheless, the acetyl-CoA-forming activity of adhE^A267T/E568K^ seemed to be quite low in *S. cerevisiae*.

**Fig. 2:**
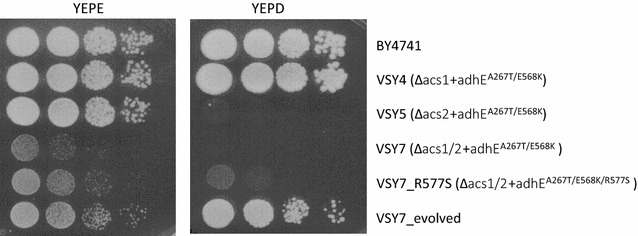
Spotting assay of adhE^A267T/E568K/R577S^ strains. Cells were grown to exponential phase and adjusted to an OD_600_ of 1. Tenfold serial dilutions were spotted onto YEPD or YEPE under aerobic conditions and incubated at 30 °C for 3 days. As a control, wild-type BY4741 (Y00000) was used

To further improve adhE^A267T/E568K^ activity, strain VSY7 (Δ*acs1/2*, *adhE* ^A267T/E568K^) was constructed which due to the deletion of both acetyl-CoA synthase genes, *ACS1* and *ACS2*, is completely dependent on adhE-mediated cytosolic acetyl-CoA production. Growth of this strain on YEPD medium was very slow but increased rapidly already after some days of incubation (see “Methods” section), indicating the occurrence of spontaneous mutations improving acetyl-CoA production. The *adhE* gene of a single clone (VSY7_evolved) was amplified and sequenced, revealing a mutation at position 1731 of the adhE coding sequence changing amino acid R577 to S. As this residue is close to mutation E568K in the ethanol oxidoreductase domain of adhE^A267T/E568K^, it was probably involved in the better growth of strain VSY7. To test this, strain VSY7_R577S was constructed by directly replacing the *adhE* ^A267T/E568K^ double-mutation allele of strain VSY7 with the *adhE* ^A267T/E568K/R577S^ triple-mutation allele. Growth was compared to the wild-type strain BY4741, the evolved VSY7 as well as VSY4 and VSY5 (Fig. 2). VSY4 is deleted for *ACS1* but still contains the constitutively expressed *ACS2,* whereas VSY5 only contains the glucose-repressed *ACS1*, and both contain the *adhE* ^A267T/E568K^ double-mutation allele. As expected, VSY4 could grow normally with glucose or ethanol and VSY5 could grow normally with ethanol. VSY5 and VSY7 exhibited only very weak growth on glucose. The *adhE* ^A267T/E568K/R577S^ triple-mutation allele clearly improved the growth of VSY7_R577S on glucose and ethanol, as compared to VSY7. Nevertheless, the original VSY7_evolved strain grew even better indicating the presence of still other mutations promoting the production of acetyl-CoA.

To directly measure the influence of the R577S mutation on the performance of adhE, the enzyme activity and stability were tested in vitro. As the enzyme activity assay is based on the oxidation of acetaldehyde and the concomitant production of NADH, a Δ*adh1*–*5* alcohol dehydrogenase-deficient yeast mutant strain had to be used to avoid the re-oxidation of produced NADH by endogenous alcohol dehydrogenases. Plasmids pVS4 (*adhE* ^A267T/E568K^), pVS4ev (*adhE* ^A267T/E568K/R577S^), and the empty vector pRS41H were transformed into strain VSY0 (Δ*adh1*–*5*), and crude extracts were prepared. AdhE^A267T/E568K^ exhibited a specific activity of 12.7 mU/mg protein, whereas adhE^A267T/E568K/R577S^ of 22.2 mU/mg, indicating that indeed the R577S mutation improved the conversion of acetaldehyde to acetyl-CoA. As Membrillo-Hernandez and coworkers [9] had found that the second mutation E568K mainly stabilizes the adhE^A267T^ single mutant form, a stability assay was performed. For that, cell extracts were incubated at 30 °C for different time periods up to 40 min. However, acetaldehyde dehydrogenase activity decreased for both, adhE^A267T/E568K^ and adhE^A267T/E568K/R577S^ (Additional file 1: Figure S5), indicating that the R577S mutation does not further stabilize the mutant adhE.

To test the effect of the *adhE* ^A267T/E568K/R577S^ triple-mutation allele on n-butanol production, strain VSY13 was constructed which is identical to VSY10 but has the *adhE* ^A267T/E568K/R577S^ triple-mutation allele instead of the *adhE* ^A267T/E568K^ double-mutation allele. In aerobic shake flask cultivation in SMD media with 25 µM pantothenate VSY13 produced slightly more n-butanol (247 mg/L) than VSY10 (Table 2). The increase was strictly dependent on the addition of pantothenate (Additional file 1: Figure S6). Also the n-butanol yield of VSY13 was increased to 0.015 g/g glucose (Table 2). The results indicate that the R577S mutation in adhE^A267T/E568K/R577S^ enables even more efficient flux from acetaldehyde to acetyl-CoA, especially in the presence of pantothenate providing more CoA [1], which is needed as the co-substrate of the mutant adhE form (Additional file 1: Figure S6).

### Enhancement of endogenous pantothenate synthesis for n-butanol production

**Fig. 3:**
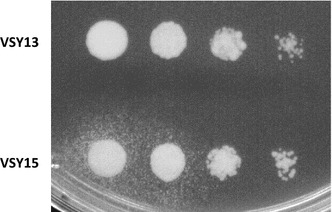
Spotting assay of pADH1_*FMS1* strains on SCD without pantothenate, containing Δ*fms1* cells. Cells were grown to exponential phase and adjusted to an OD_600_ of 1. Tenfold serial dilutions were spotted onto SCD lacking pantothenate, whereby 10 µl of Δ*fms1* overnight culture was added to the medium. Control VSY13 (Δ*adh1*–*6 sfa1 gpd2*, n-butanol pathway genes, *coaA* and *adhE* ^A267T/E568K/R577S^) and VSY15 (VSY13 with pADH1_*FMS1*) were compared. Cells were incubated aerobically at 30 °C for 4 days

We had shown before that overexpression of pantothenate kinase coaA and addition of pantothenate increased n-butanol production due to an increased supply of CoA [2]. However, pantothenate is expensive and its addition is not economical under industrial conditions. Therefore, we reasoned to improve its endogenous production. Pantothenate is synthesized by condensation of β-alanine and pantoate (Fig. 1). β-Alanine is derived from spermine via Fms1 and Ald2/3, whereas pantoate is derived from 2-ketoisovalerate via Ecm31 and Pan5. White and coworkers [14] have shown that overexpression of amine oxidase Fms1 leads to overproduction of pantothenate and its secretion into the medium, indicating Fms1 as a limiting step in the pathway. To test whether this is true also for our strains, the native promoter of *FMS1* was replaced by the strong *ADH1* promoter in the genome of VSY13, resulting in strain VSY15. A spotting assay was performed with Δ*fms1* pantothenate auxotrophic cells, which were included in SCD agar medium without pantothenate (Fig. 3). Whereas VSY13 cells did not support the growth of the Δ*fms1* cells, halos of growing Δ*fms1* cells could be observed surrounding the spots with VSY15 cells. This indicated that in contrast to VSY13, the VSY15 cells overproduce and even secrete a compound that can complement the pantothenate auxotrophy downstream of Fms1. As also Δ*ecm31* mutant cells [14] and Δ*pan6* cells (data not shown) could be complemented by spots of *FMS1* overexpressing cells, it is likely that the secreted compound is pantothenate. Secretion of pantothenate by VSY15 was a little bit surprising as VSY15 overexpresses pantothenate kinase coaA. Therefore, the results indicate that this reaction or a reaction further downstream in the CoA pathway is still limiting CoA synthesis. Nevertheless, the results demonstrate that the overexpression of *FMS1* resulted in overproduction of pantothenate. On the other hand, overexpression of *ALD2* and/or *PAN6* did not lead to the secretion of pantothenate and did not even further increase the diameter of Δ*fms1* cell halos in combination with *FMS1* overexpression (data not shown).

In order to test the effect of endogenous pantothenate overproduction on n-butanol formation, aerobic fermentations were performed with VSY15 in SMD medium without pantothenate. A significantly higher n-butanol titer was produced by VSY15 (243 mg/L) compared to its progenitor VSY13 (165 mg/L) (Table 2). Interestingly, the n-butanol titer produced by VSY15 in the absence of pantothenate was nearly exactly the same as the titer of VSY13 in the presence of additional panthothenate (247 mg/L), indicating that overexpression of *FMS1* can completely replace the addition of exogenous pantothenate.

### The trans-2-enoyl-CoA reductase (ter) reaction is a bottleneck in the acetoacetyl-CoA-derived n-butanol pathway

**Fig. 4:**
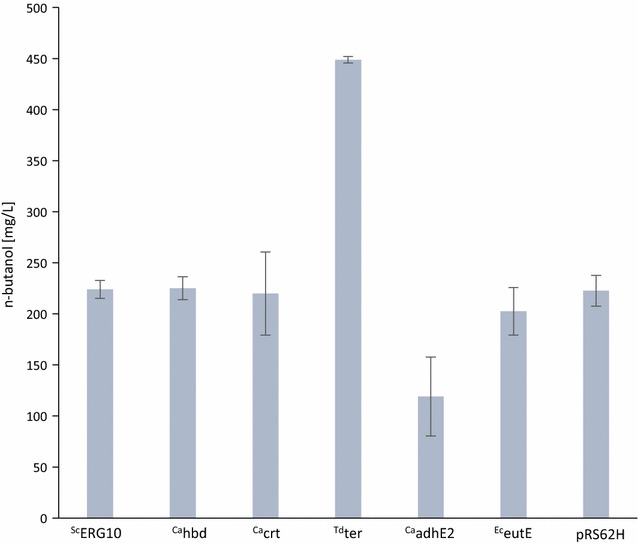
n-Butanol concentrations of n-butanol-producing yeast strains additionally overexpressing enzymes of the n-butanol biosynthesis pathway. Shown are maximum n-butanol titers (^Ca^ *hbd* and ^Td^ *ter* after 74 h and others after 100 h) of aerobic fermentations in SMD media. Bottleneck analysis was carried out with VSY15 (Δ*adh1*-*6 sfa1 gpd2* with n-butanol pathway genes, *coaA* and *adhE* ^A267T/E568K/R577S^, pADH1_*FMS1*) overexpressing pRS62H (high copy vector, promoter pHXT7, terminator tFBA1) with one each enzyme of n-butanol pathway. As a control, empty vector pRS62H was used. Genes from Sc: *Saccharomyces cerevisiae*, Ca: *Clostridium acetobutylicum*, Ec: *E.coli*, and Td: *Treponema denticola* are indicated by prefixes in superscript. *Error bars* represent the standard deviation of three independent replicates

An additional copy of *adhE* ^A267T/E568K/R577S^ by transformation of VSY15 with plasmid pVS4ev only marginally increased n-butanol production (254 mg/L) (Table 2). Therefore, it was reasonable to test whether a bottleneck in the heterologous n-butanol synthesis pathway might limit n-butanol production. To test this, all the genes of the n-butanol pathway were individually overexpressed from multi-copy vectors in strain VSY15 in addition to the genomic copies. The genes ^Sc^ *ERG10*, ^Ca^ *hbd*, ^Ca^ *crt*, ^Td^ *ter*, ^Ec^ *eutE,* and ^Ca^ *adhE2* were each cloned on a high copy vector (pRS62H) behind the strong *HXT7* promoter fragment, transformed into VSY15, and aerobic fermentations were performed in SMD medium with hygromycine. The control strain with the empty vector reached n-butanol titers of 223 mg/L (Fig. 4). Strains overexpressing *ERG10, hbd, crt,* or *eutE* reached similar levels. Overexpression of ^Ca^ *adhE2* had a negative effect on growth and n-butanol production. Interestingly, overexpression of ^Td^ *ter* had a positive effect on n-butanol production (Fig. 4) and the n-butanol titer, and the yield increased up to 449 mg/L and 0.028 g/g glucose, respectively (Table 2). These results indicate that the reduction of crotonyl-CoA into butyryl-CoA by ter is a limiting step in the pathway. They are consistent with the low enzyme activities of ter in yeast [2] and the secretion of crotonal, probably derived from crotonyl-CoA, as observed by Swidah and coworkers [25].

A block in the reductive part of n-butanol production might lead to inefficient NADH re-oxidation and therefore an insufficient supply of NAD^+^ for glycolysis and the acetylating adhE. Therefore, we tested whether expression of a water-forming soluble oxygen-dependent NADH oxidase (nox) from *Streptococcus pneumoniae* [26] might be beneficial for growth, glucose consumption, and product formation. For this, VSY15 was transformed with the low copy plasmid pRS41H-pTPI1_NOX, which carries a codon-optimized version of nox expressed behind the strong *TPI1* promoter and as a control with the empty vector pRS41H. Indeed, in aerobic fermentations, growth and glucose consumption was increased especially in the beginning of the fermentation (Additional file 1: Figure S7). However, n-butanol production was significantly reduced (89 mg/L compared to 225 mg/L). These results indicate that the availability of NAD^+^ might be important for the growth performance of the cells, but that high levels of NADH are needed as a driving force for the n-butanol production. Obviously, the expression level of NADH oxidase would need to be adjusted very precisely in order to balance the optimal NAD^+^/NADH ratios.

### Deletion of acetaldehyde dehydrogenase *ALD6* gene further improves n-butanol production

In VSY15, acetaldehyde can be converted to acetyl-CoA either via the adhE^A267T/E568K/R577S^ or via acetaldehyde dehydrogenases (mainly Ald6) followed by Acs1/2 acetyl-CoA synthetases. In the Ald6-dependent pathway, acetate is an intermediate, and can be lost out of the cells. Moreover, adhE produces NADH, while Ald6 produces NADPH. As the n-butanol pathway is strictly dependent on NADH, the adhE pathway should be more favorable for n-butanol production. *S. cerevisiae* does not contain transhydrogenases, and therefore NADPH and NADH cannot be converted into one another [27]. However, in VSY15, adhE has to compete with Ald6 for the substrate acetaldehyde. Also, the Ald6-dependent pathway is energetically more costly because acetyl-CoA synthetases hydrolyze ATP to AMP and pyrophosphate, whereas adhE does not use ATP.

To strengthen the adhE pathway, we deleted *ALD6* in strain VSY15, resulting in VSY19, and performed aerobic fermentations in SMD medium (﻿Fig. ﻿5). Indeed, n-butanol production increased remarkably from 243 mg/L (strain VSY15) to 634 mg/L with strain VSY19 (Table 2). The n-butanol yield increased from 0.016 to 0.057 g/g glucose. The deletion of *ALD6* was also reflected in lower acetate production (432 mg/L in VSY15 and 178 mg/L in VSY19). An additional copy of *adhE* ^A267T/E568K/R577S^ by transformation of VSY19 with plasmid pVS4ev only marginally increased n-butanol production (643 mg/L) (Table 2). However, additional overexpression of ter by transformation of VSY19 with plasmid pRS62H_tdTer further increased the n-butanol titer to 859 mg/L and the yield to 0.071 g/g glucose. The results show that adhE^A267T/E568K/R577S^ can convert acetaldehyde more efficiently into acetyl-CoA if the competing NADP^+^- and ATP-dependent pathway are blocked. This prevents the leakage of acetate and favors formation of NADH as a driving force for n-butanol production.

**Fig. 5:**
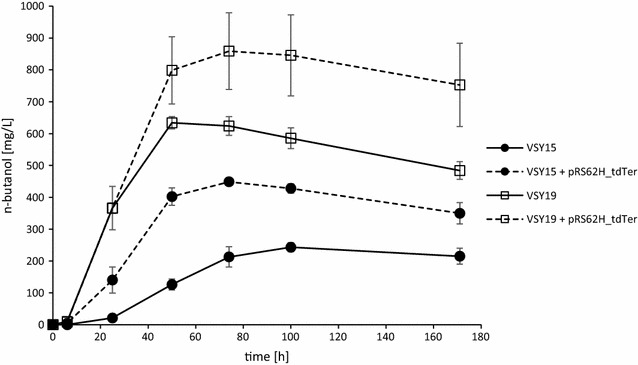
n-Butanol production of VSY15 and VSY19 with or w/o additional overexpression of ter. n-Butanol titers are shown of VSY15 (Δ*adh1*–*6 sfa1 gpd2* with n-butanol pathway genes, *coaA*, *adhE* ^A267T/E568K/R577S^, pADH1_*FMS1*) and VSY19 (VSY15 with Δ*ald6*) with or without pRS62H_tdTer. *Error bars* represent the standard deviation of three independent replicates

## Conclusion

One of the major problems in n-butanol production with yeast seems to keep an optimal redox balance of the cofactor NAD between its oxidized form, NAD^+^, and the reduced form, NADH. Glycolysis is very fast in yeast, and therefore there is a high need for NAD^+^. On the other hand, the driving force for high production rates of n-butanol is NADH [16]. But, obviously flux through the n-butanol pathway in yeast is not yet high enough to provide enough NAD^+^ for the continuation of glycolysis. This finally results in a slowdown of glucose consumption. Expression of nox could in fact partially alleviate glucose consumption by increasing NADH re-oxidation, but then obviously NADH was lacking as driving force for n-butanol production. In contrast, the aerobic conditions employed in this work, probably by allowing some NAD^+^ regeneration, had a positive effect on both glucose consumption and n-butanol formation. Moreover, blocking NADPH formation by deletion of *ALD6* in favor of NADH formation via adhE strongly improved n-butanol production. On the other hand, provision of an excess supply of NADH using the more reduced sugar alcohol sorbitol as the carbon source after overexpression of a sorbitol dehydrogenase and a sorbitol transporter [28] in the n-butanol-producing strain VSY19 had a detrimental effect on growth, sorbitol consumption, and butanol production (data not shown) in contrast to the fast growth and sorbitol consumption of a wild-type ethanol-producing yeast strain [28].

The limiting step in the n-butanol pathway seems to be the ter reaction. Indeed, this was already indicated by the work of Swidah and coworkers [25] who observed crotonal secretion in n-butanol-producing yeasts. Therefore, there is a high need to optimize the conversion of crotonyl-CoA to butyryl-CoA for improved n-butanol production with yeast.

The other driving force for n-butanol production is acetyl-CoA. In this work, we could further improve its synthesis by providing more substrate in the form of CoA via endogenous overproduction of pantothenate. Moreover, we found an improved version of acetylating acetaldehyde dehydrogenase adhE, adhE^A267T/E568K/R577S^. Nevertheless, although the pantothenate kinase reaction was already improved, there still seems to be a bottleneck in the CoA biosynthesis pathway as pantothenate was even secreted out of the cells. Moreover, as indicated by the poor complementation of *acs* mutants, also adhE seems to be not yet working optimally.

## Additional file

**Additional file 1: Figure S1.** Metabolic pathway for n-butanol production via reverse β-oxidation in yeast. **Figure S2.** Comparison of glucose consumption and final OD_600_ of n-butanol production strains. **Figure S3.** Comparison of ethanol concentration of n-butanol production strains. **Figure S4.** Enzymatic reactions of adhE from *E.coli.* **Figure S5.** Stability assay of adhE^A267T/E568K/R577S^. **Figure S6.** n-Butanol production of VSY13 under aerobic conditions with addition of pantothenate. **Figure S7.** Aerobic fermentation of VSY15 containing NADH oxidase nox from *Streptococcus pneumoniae.* **Table S1** Relevant primers for this study. **Table S2.** Yeast promoters and terminators used for the expression of n-butanol pathway genes and endogenous pantothenate synthesis in this study. **Table S3.** Statistical analysis of n-butanol production.

## Abbreviations

OD_600_: optical density at 600 nm
SMD: synthetic minimal medium containing glucose
ADH: alcohol dehydrogenase
ter: trans-2-enoyl-CoA reductase
