# Supplementary material for: Increasing n-butanol production with Saccharomyces cerevisiae by optimizing acetyl-CoA synthesis, NADH levels and trans-2-enoyl-CoA reductase expression
Source: Biotechnol Biofuels. 2016 Nov 25;9:257. doi: 10.1186/s13068-016-0673-0 (PMC5123364; doi:10.1186/s13068-016-0673-0)
Supplement: Supplementary file 1 — Additional file 1: Figure S1. Metabolic pathway for n-butanol production via reverse β-oxidation in yeast. Figure S2. Comparison of glucose consumption and final OD600 of n-butanol production strains. Figure S3. Comparison of ethanol concentration of n-butanol production strains. Figure S4. Enzymatic reactions of adhE from E.coli. Figure S5. Stability assay of adhEA267T/E568K/R577S. Figure S6. n-Butanol production of VSY13 under aerobic conditions with addition of pantothenate. Figure S7. Aerobic fermentation of VSY15 containing NADH oxidase nox from Streptococcus pneumoniae. Table S1 Relevant primers for this study. Table S2. Yeast promoters and terminators used for the expression of n-butanol pathway genes and endogenous pantothenate synthesis in this study. Table S3. Statistical analysis of n-butanol production. [file 13068_2016_673_MOESM1_ESM.pdf]

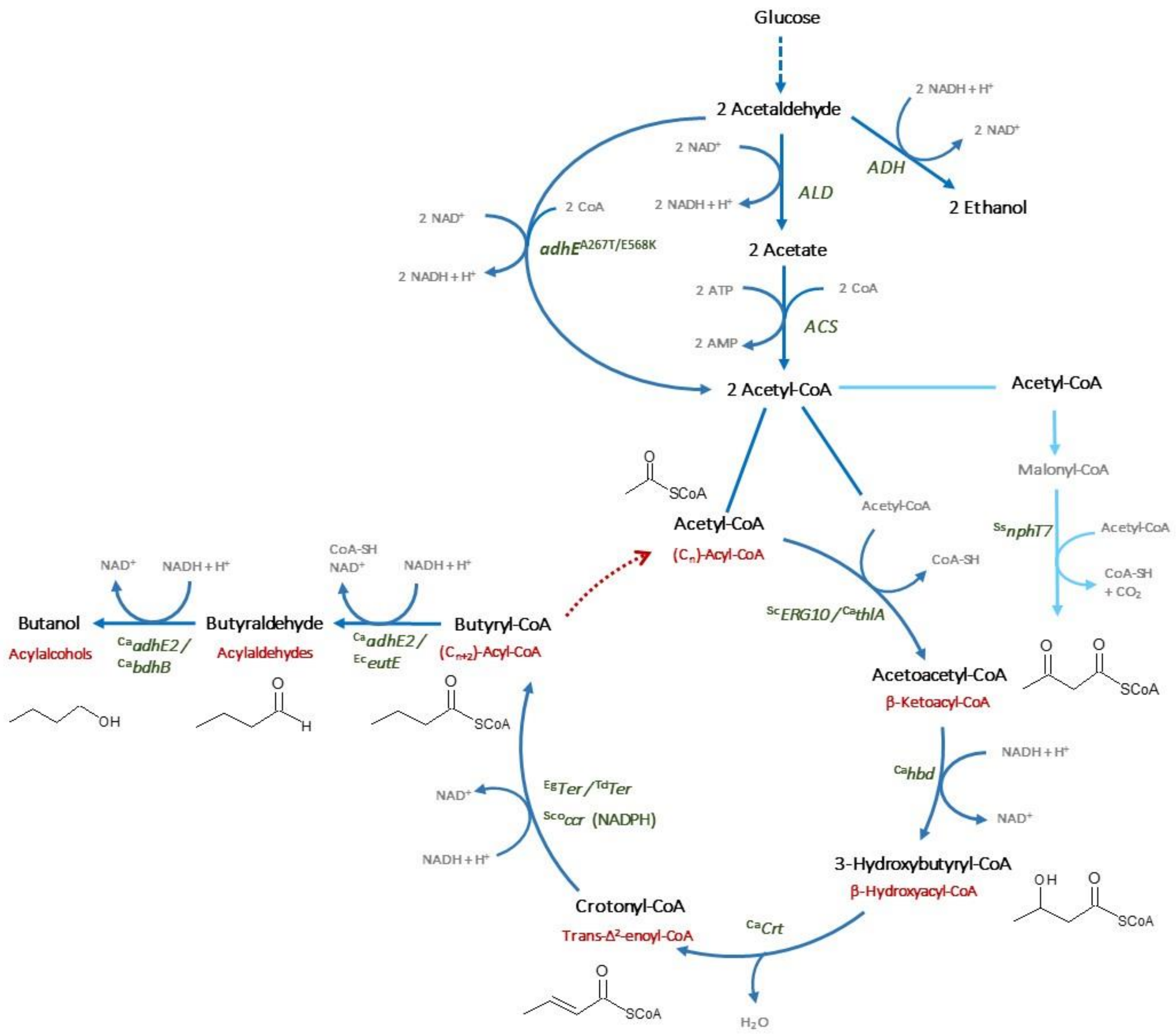

Figure S1: Metabolic pathway for n-butanol production via reverse β-oxidation in yeast.
n-Butanol production and reverse β-oxidation are shown as a process of one or several rotations, respectively, of an acetoacetyl-CoA derived synthesis pathway. This pathway can generate various compounds with different chain lengths. The different isoenzymes, tested in the former study [1], are shown for each reaction. The reaction of [Ss]*nphT7* (use of malonyl-CoA and acetyl-CoA for production of acetoacetyl-CoA) is shown in light blue [1].

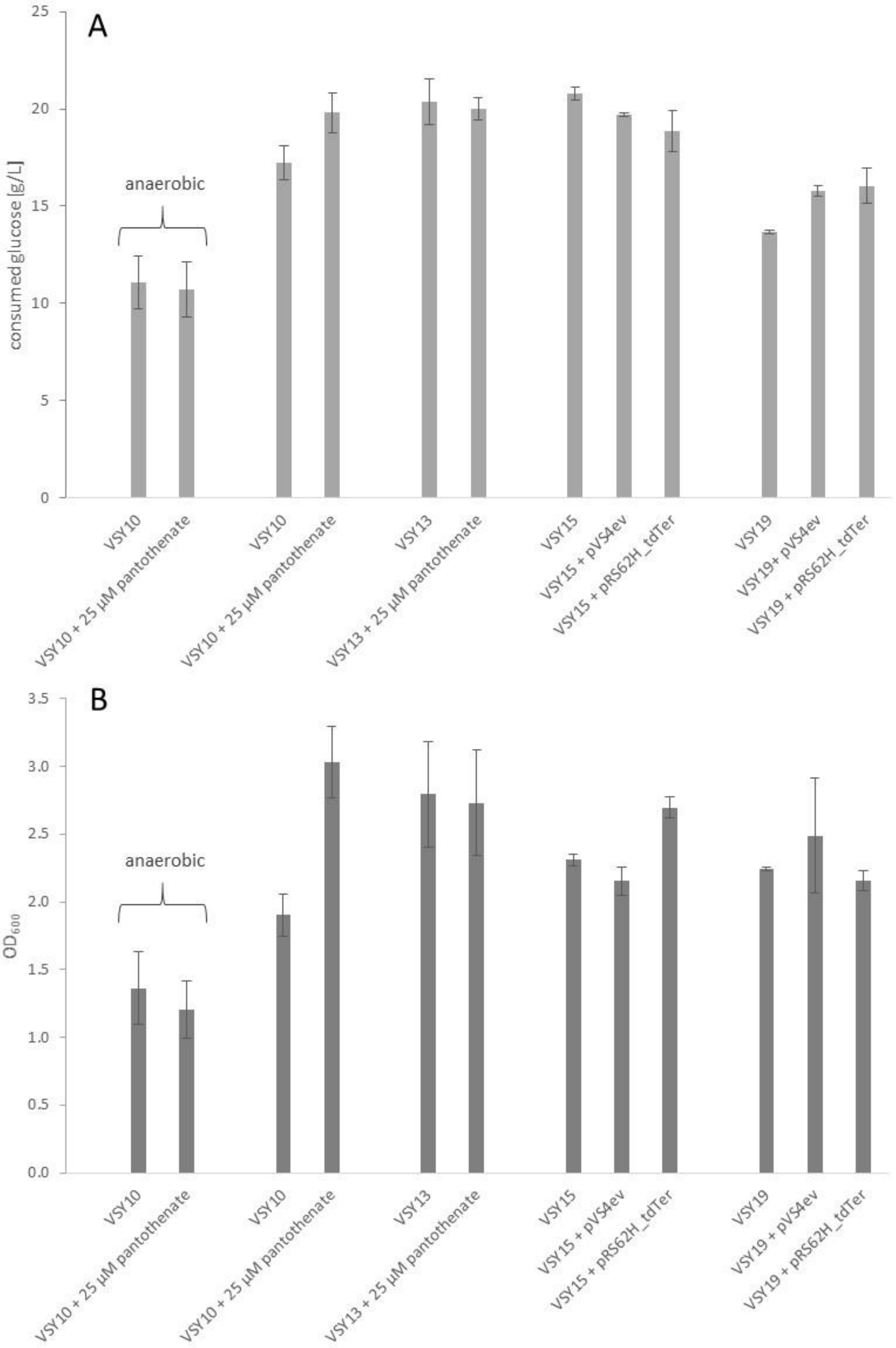

Figure S2: Comparison of glucose consumption and final OD_{600} of n-butanol production strains.
A Consumed glucose after 171 h of fermentation started with 20 g/L glucose in SMD medium. **B** The OD_{600} after 171 h of fermentations started with an OD_{600} of 0.3 in SMD medium.
Compared are fermentations of VSY10 (Δ*adh1-6 sfa1 gpd2* with n-butanol pathway genes, *coaA* and *adhE*$^{A267T/E568K}$) anaerobically and aerobically, VSY13 (like VSY10, but *adhE*$^{A267T/E568K/R577S}$ instead of *adhE*$^{A267T/E568K}$), VSY15 (VSY13 with pADH1_*FMS1*) and VSY19 (VSY15 with Δ*ald6*) with or without addition of 25 µM pantothenate or *adhE*$^{A267T/E568K/R577S}$ (pVS4ev) or pRS62H_tdTer overexpression. Error bars represent the standard deviation of three independent replicates.

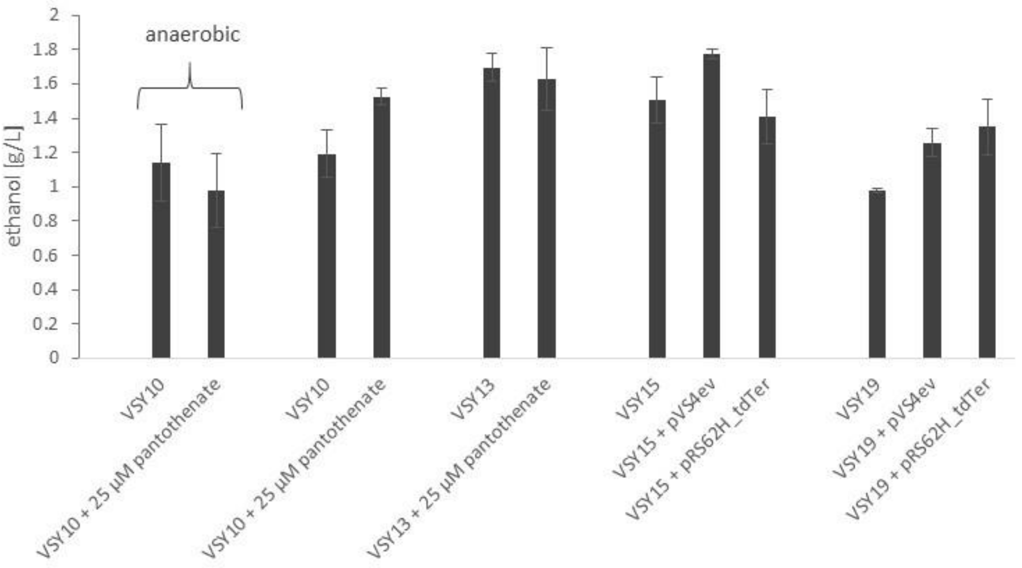

Figure S3: Comparison of ethanol concentration of n-butanol production strains.
The ethanol concentration after 171 h of fermentations started with an OD_{600} of 0.3 in SMD medium is shown. Compared are fermentations of VSY10 (Δ*adh1-6 sfa1 gpd2* with n-butanol pathway genes, *coaA* and *adhE*$^{A267T/E568K}$) anaerobically and aerobically, VSY13 (like VSY10, but *adhE*$^{A267T/E568K/R577S}$ instead of *adhE*$^{A267T/E568K}$), VSY15 (VSY13 with pADH1_*FMS1*) and VSY19 (VSY15 with Δ*ald6*) with or without addition of 25 µM pantothenate or *adhE*$^{A267T/E568K/R577S}$ (pVS4ev) or pRS62H_tdTer overexpression. Error bars represent the standard deviation of three independent replicates.

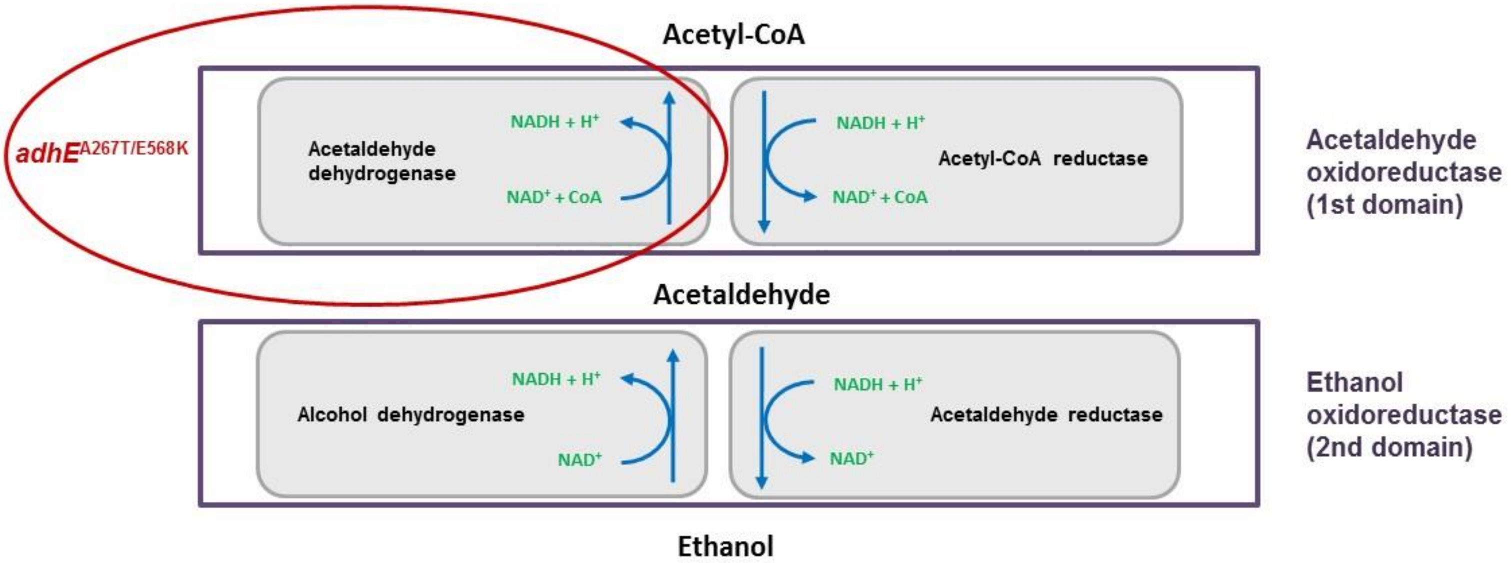

Figure S4: Enzymatic reactions of adhE from *E.coli*
Shown are possible reaction steps of multifunctional oxidoreductase adhE. With the wild type enzyme reduction from acetyl-CoA into acetaldehyde into ethanol takes place, but with amino acid changes A267T and E568K, adhE preferably converts acetaldehyde into acetyl-CoA [2].

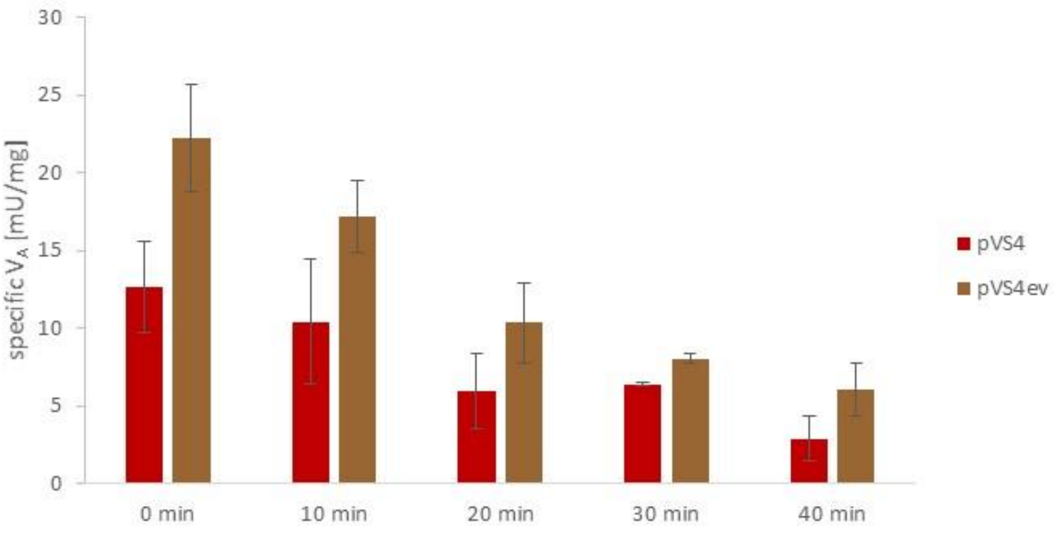

Figure S5: Stability assay of adhE$^{A267T/E568K/R577S}$.

Dehydrogenase activity of acetylating acetaldehyde dehydrogenase *adhE*$^{A267T/E568K}$ (pVS4) and *adhE*$^{A267T/E568K /R577S}$ (pVS4ev) was measured directly after incubation of cell extract for 0 to 40 min at 30° (based on [2]). One unit (U) is defined as the conversion of 1 µmol of substrate into the corresponding product per 1 min. The mean values of three independent replicates are shown with standard deviations.

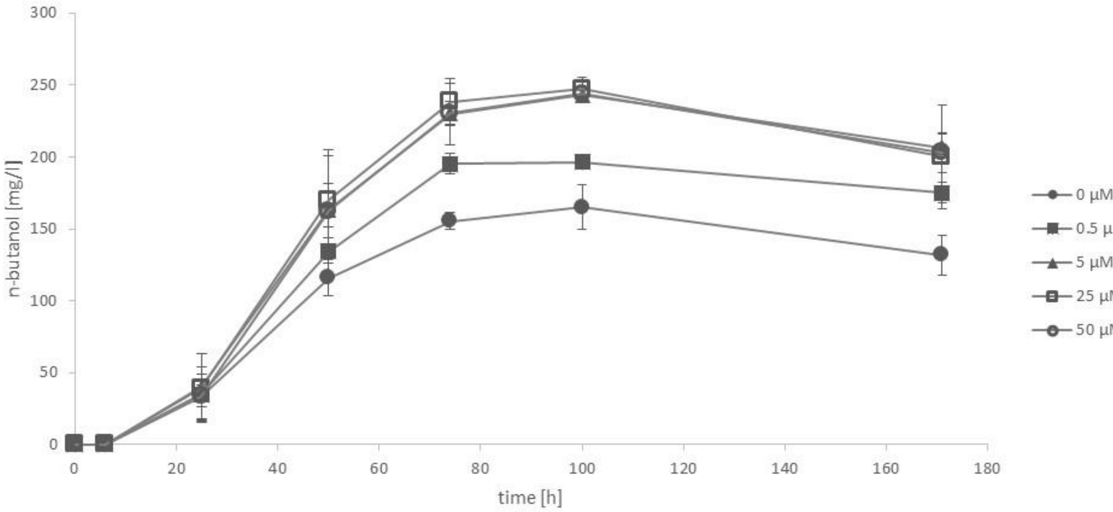

Figure S6: n-Butanol production of VSY13 under aerobic conditions with addition of pantothenate. Five different concentrations of pantothenate were added to SMD medium: 0 µM (circle), 0.5 µM (square), 5 µM (triangle), 25 µM (open square), 50 µM (open circle). Error bars represent the standard deviation of three independent replicates.

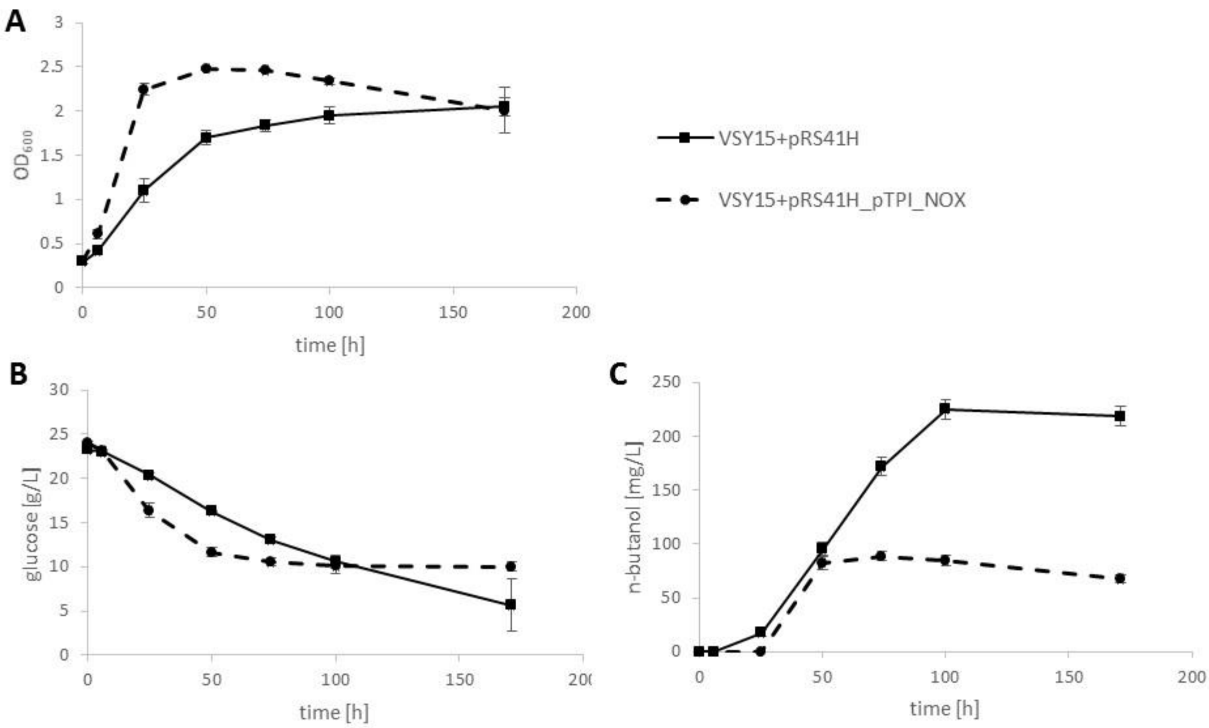

Figure S7: Aerobic fermentation of VSY15 containing NADH oxidase nox from *Streptococcus pneumoniae.*

Shown are values of OD_{600} (A), glucose (B) and n-butanol (C) concentration of fermentations in SMD media with VSY15 (Δ*adh1-6 sfa1 gpd2*, n-butanol pathway genes, *coaA* and $adhE^{A267T/E568K/R577S}$, pADH1_*FMS1*) with pRS41H_pTPI_NOX (dashed line) or empty vector pRS41H as a control (solid line).

Table S1: Relevant primers for this study
The abbreviations within the primer names were used as follows: forward primer (fw), reverse primer (rev), overlap (ov). Other primers for construction of all strains and plasmids are available in [2].

Primer name	Sequence 5'-3'	Description
vsp329_pVS4-Kass_ovtACS2_rev	ATTACGAAATTTTTCTCATTTAAGGAAAAATATAAG GATGAGAAAGTGAAATCGG	fusion-PCR for *adhE* $^{A267T/E568K/R577S}$ in *ACS2*
vsp330_pVS4-Kass_ovpACS2_fw	AGAATACAGGAAAGTAAATCAATACAATAATAAAA TCAGGCCTAGGCGACAACCC	fusion-PCR for *adhE* $^{A267T/E568K/R577S}$ in *ACS2*
vsp331_pACS2_ovpVS4-Kass_rev	GGGTTGTCGCCTAGGCCTGATTTTATTATTGTATTG ATTTACTTTCC	fusion-PCR for *adhE* $^{A267T/E568K/R577S}$ in *ACS2*
vsp332_tACS2_ovpVS4-Kass_fw	TCACTTTCTCATCCTTATATTTTTCCTTAAATGAGAA AAATTTCGTAATG	fusion-PCR for *adhE* $^{A267T/E568K/R577S}$ in *ACS2*
vsp333_tACS2_rev	AACAAGGCAAAATAGCGTTAACAACC	verification chr. integration of *adhE*
vsp334_pACS2_fw	TTTCCTGTGAGAAGTTTAAATCCACTAAGG	verification chr. integration of *adhE*
vsp335_ACS1_rev	GGTGCGGCAAATGGCCCG	test-PCR within gene (A6) for *ACS1*
vsp336_ACS1_fw	GGGCGTTCGCAAGGGCG	test-PCR within gene (A5) for *ACS1*
vsp337_ACS2_rev	GGCAGGTACAACTTCTGGGTTGGCC	test-PCR within gene (A6) for *ACS2*
vsp338_ACS2_fw	TTGGCTGTGGCTCGTATTGGTGC	test-PCR within gene (A5) for *ACS2*
vsp339_SFA1_rev	CACCAGCGGCAGCCACACC	test-PCR within *SFA1*
vsp340_SFA1_fw	GGGCCACGAAGGAGCCGG	test-PCR within *SFA1*
vsp368_CC-ACS1_fw	CCAAGACGTGTCTCACGCCTGTTTTAGAGCTAGAAA TAGCAAGTTAAAATAAGG	CrisprCas for *ACS1* deletion
vsp368_CC-ACS1_rev	AGGCGTGAGACACGTCTTGGGATCATTTATCTTTCA CTGCGGAG	CrisprCas for *ACS1* deletion
vsp370_CC-ACS1-Donor	TCCTCCCAGAAAAACAATCTGTTTATTACCCGACAT TCTTCACTTTATGGGCAGTCCTTGTATCGACTTGCTC ATTATAA	CrisprCas for *ACS1* deletion
vsp371_pFMS1_ovtHis3_rev	ATTTTTATAGGTTAATGTCATGCTTATATACAATATA ATAATATGTAGGTAAACGTG	fusion-PCR for pADH1-*FMS1*
vsp372_pFMS1_fw	AATAGAAATAAGTTTGAGCAGTTCAAG	fusion-PCR for pADH1-*FMS1*
vsp373_tHis3_ovtADH1_rev	CCCCCTTCTCTACTAGCATTGGACTTAATGAGCTGA TTTAACAAAAATTTAACGC	fusion-PCR for pADH1-*FMS1*
vsp374_pHis3_ovpFMS1_fw	ACCTACATATTATTATATTGTATATAAGCATGACATT AACCTATAAAAATAGGCG	fusion-PCR for pADH1-*FMS1*
vsp375_pADH1_ovFMS1_rev	TTTGGCTGGTGAAACTGTATTCATTGTATATGAGAT AGTTGATTGTATGC	fusion-PCR for pADH1-*FMS1*
vsp376_pADH1_ovtHis3_fw	AAATTTTTGTTAAATCAGCTCATTAAGTCCAATGCT AGTAGAGAAGG	fusion-PCR for pADH1-*FMS1*
vsp377_FMS1_rev	CTAAGTGTTGATGGAATTCTAACTCTG	fusion-PCR for pADH1-*FMS1*
vsp378_FMS1_ovpADH1_fw	GCATACAATCAACTATCTCATATACAATGAATACAG TTTCACCAGCC	fusion-PCR for pADH1-*FMS1*
vsp379_FMS1_rev	GGCCTCATTCCGTCGATGACATCC	verification fusion-PCR for pADH1-*FMS1*
vsp380_pFMS1_fw	CTGCACGCAGGGATTGCCG	verification fusion-PCR for pADH1-*FMS1*
vsp388_CC-ALD6_fw	AAAACTTTGGCCTTAGCCCGGTTTTAGAGCTAGAAA TAGCAAGTTAAAATAAGG	CrisprCas for deletion of *ALD6*
vsp389_CC-ALD6_rev	CGGGCTAAGGCCAAAGTTTTGATCATTTATCTTTCA CTGCGGAG	CrisprCas for deletion of *ALD6*
vsp390_CC-ALD6-Donor	AACATCTTTAACATACACAAACACATACTATCAGAA TACATGTACCAACCTGCATTTCTTTCCGTCATATACA CAAAATA	CrisprCas for deletion of *ALD6*

vsp391_tALD6_rev	GCGAAATGGCAGTACTCGGGGG	verification *ALD6*-deletion
vsp392_pALD6_fw	TCGTAATAAATTCGGGGTGAGGGGG	verification *ALD6*-deletion
vsp393_ALD6_rev	TACCGGCCTTCAACATCTTGGCC	test-PCR within gene (A5) for *ALD6*
vsp394_ALD6_fw	TCCACGACACTGAATGGGCTACCC	test-PCR within gene (A6) for *ALD6*
vsp398_ERG10_ovpHXT7_fw	AAACACAAAAACAAAAAGTTTTTTTAATTTTAATCA AAAAATGTCTCAAAACGTTTACATTG	bottleneck-analysis
vsp399_ERG10_ovtFBA1_rev	AATACTCATTAAAAAACTATATCAATTAATTTGAATT AACTTAAATCTTTTCAATGACAATAGAGG	bottleneck-analysis
vsp400_hbd_ovpHXT7_fw	AAACACAAAAACAAAAAGTTTTTTTAATTTTAATCA AAAAATGAAGAAGGTTTGTGTTATTGG	bottleneck-analysis
vsp401_hbd_ovtFBA1_rev	AATACTCATTAAAAAACTATATCAATTAATTTGAATT AACTTACTTAGAGTAATCGTAGAAACCC	bottleneck-analysis
vsp402_crt_ovpHXT7_fw	AAACACAAAAACAAAAAGTTTTTTTAATTTTAATCA AAAAATGGAATTGAACAACGTCATC	bottleneck-analysis
vsp403_crt_ovtFBA1_rev	AATACTCATTAAAAAACTATATCAATTAATTTGAATT AACTTATCTGTTCTTGAAACCTTCAATC	bottleneck-analysis
vsp404_tdTer_ovpHXT7_fw	AAACACAAAAACAAAAAGTTTTTTTAATTTTAATCA AAAAATGATTGTTAAGCCAATGGTTAGAAACAAC	bottleneck-analysis
vsp405_tdTer_ovtFBA1_rev	AATACTCATTAAAAAACTATATCAATTAATTTGAATT AACTTAAATTCTGTCGAATCTTTCAACTTCAGC	bottleneck-analysis
vsp406_adhE2_ovpHXT7_fw	AAACACAAAAACAAAAAGTTTTTTTAATTTTAATCA AAAAATGAAGGTTACCAACCAAAAG	bottleneck-analysis
vsp407_adhE2_ovtFBA1_rev	AATACTCATTAAAAAACTATATCAATTAATTTGAATT AACTTAGAAAGACTTAATGTAGATATCCTTC	bottleneck-analysis
vsp408_eutE_ovpHXT7_fw	AAACACAAAAACAAAAAGTTTTTTTAATTTTAATCA AAAAATGAACCAACAAGATATTGAACAAGTTG	bottleneck-analysis
vsp409_eutE_ovtFBA1_rev	AATACTCATTAAAAAACTATATCAATTAATTTGAATT AACTTAAACAATTCTGAAAGCATCGACC	bottleneck-analysis

Table S2: Yeast promoters and terminators used for the expression of n-butanol pathway genes and endogenous pantothenate synthesis in this study. DNA sequences were amplified from chromosomal DNA of CEN.PK2-1C. When not otherwise indicated, promoters were taken from 1-500 bps before and terminators 1-300 bps behind the respective open reading frames. Either all fragments were integrated into the chromosome via an integration cassette or introduced via vectors (in case of ^{Sc}*ALD2* and ^{Sc}*PAN6*), except for *FMS1*, whose promoter was exchanged in the genome. Genes from *Saccharomyces cerevisiae* (Sc), *Clostridium acetobutylicum* (Ca), *E.coli* (Ec), *Treponema denticola* (Td) are indicated by prefixes in superscript.

promoter	gene	terminator
*pHXT7**	^{Sc}*ERG10*	*tVMA16*
pPGK1	^{Ca}*hbd*	*tEFM1*
pTPI1	^{Ca}*crt*	*tYHI9*
pPYK1	^{Td}*ter*	*tIDP1*
*pADH1***	^{Ca}*adhE2*	*tRPL3*
pTDH3	^{Ec}*eutE*	*tRPL41B*
pPFK1	^{Ec}*adhE* $^{A267T/E568K/R577S}$	*tDIT1*
*pPMA1****	^{Ec}*coaA*	*tVMA2*
*pADH1***	^{Sc}*FMS1*	*tFMS1*
pFBA1	^{Sc}*ALD2*	*tPRC1*
*pGPM1*****	^{Sc}*PAN6*	*tNAT5*

* (-1 to -389 bp; [3])
** (-1 to -800 bp; [4])
*** (-1 to-575 bp, -700 to -925 bp; [5])
**** (-1 to -395 bp; [6])

Table S3: Statistical analysis of n-butanol production

A t-test was performed (unpaired, two-tailed, confidence: 95%) with program GraphPad Prism 5. n-Butanol titers of strains without or with additional adhE triple mutant (pVS4ev and VSY13 with genomic integration of *adhE*$^{A267T/E568K/R577S}$) of table 2 were compared (strain 1 with strain 2).

Strain 1	Strain 2	p-value of titer
VSY10	VSY13	0.24
VSY15	VSY15 + pVS4ev	0.27
VSY19	VSY19 + pVS4ev	0.57

References

1. Schadeweg V, Boles E. n-Butanol production in *Saccharomyces cerevisiae* is limited by the availability of coenzyme A and cytosolic acetyl-CoA. Biotechnol Biofuels. 2016;9:44.
2. Membrillo-Hernandez J, Echave P, Cabiscol E, Tamarit J, Ros J, Lin ECC. Evolution of the *adhE* gene product of *Escherichia coli* from a functional reductase to a dehydrogenase. Genetic and biochemical studies of the mutant proteins. J Biol Chem. 2000;275:33869–33875.
3. Becker J, Boles E. A modified *Saccharomyces cerevisiae* strain that consumes L-arabinose and produces ethanol. Appl Environ Microbiol. 2003;69:4144–4150.
4. Tornow J, Santangelo GM. Efficient expression of the *Saccharomyces cerevisiae* glycolytic gene *ADH1* is dependent upon a cis -acting regulatory element (UASRPG) found initially in genes encoding ribosomal proteins. Gene. 1990;90:79–85.
5. Capieaux E, Vignais ML, Sentenac A, Goffeau A. The yeast H+-ATPase gene is controlled by the promoter binding factor TUF. J Biol Chem 1989;264:7437-7446.
6. Rodicio R, Heinisch JJ, Hollenberg CP. Transcriptional control of yeast phosphoglycerate mutase-encoding gene. Gene. 1993;125:125–133.
